# Sociodemographic patterns of provider-to-home telehealth use within the Veterans Health Administration between 2015 and 2023

**DOI:** 10.1007/s44250-025-00256-0

**Published:** 2025-07-09

**Authors:** Navid Dardashti, Jacqueline M. Ferguson, Andrew Nicholson, Leonie Heyworth, Timothy P. Hogan, Nicholas McMahon, Cindie Slightam, Donna M. Zulman, Scott E. Sherman

**Affiliations:** 1 Virtual Care Consortium of Research, VA New York Harbor Healthcare System, 423 E 23rd St. 15 North, New York, NY 10010 USA; 2 NYU Grossman School of Medicine, New York, NY USA; 3 VA Center for Innovation to Implementation (Ci2i), Menlo Park, CA USA; 4 Division of Primary Care and Population Health, Stanford University School of Medicine, Stanford, CA USA; 5 Department of Veterans Affairs Central Office, Office of Connected Care/Telehealth, Washington, DC USA; 6 Center for Healthcare Organization and Implementation Research, VA Bedford Healthcare System, Bedford, MA USA; 7 Peter O’Donnell School of Public Health, UT Southwestern Medical Center, Dallas, TX USA

**Keywords:** Veterans, Video care, Telemedicine, Telehealth, Provider-to-home

## Abstract

**Background:**

The VHA is the largest healthcare system in the US and an early adopter of telehealth. Barriers to adoption may exist among subpopulations of VHA patients.

**Objective:**

To identify patterns in use of telehealth by modality, race, rurality, age and priority group before and during the COVID-19 pandemic.

**Design:**

We used data from the VHA Pyramid Analytics database to determine quarterly telehealth utilization rates from October 2015 to March 2023 using a pre-post analysis. Main measures were stratified by race, rurality, age group, and VA priority groups.

**Participants:**

Unique patients who used any VHA care within each Fiscal Year of the study period.

**Interventions:**

N/A.

**Main Measures:**

Quarterly encounters by modality and number of users with one or more Provider to Home (PTH) encounters per 1000 unique patients.

**Key Results:**

There were 36,315,299 telehealth encounters completed by 4,597,055 users during the analytic period. From October 2015–March 2020, PTH video encounters grew from 3.2% of VHA telehealth encounters to 38%. From April 2020–March 2023, PTH video encounters accounted for 90.7% of VHA telehealth encounters. Uptake of PTH during the pandemic differed significantly between demographic groups. Quarterly users per 1000 unique patients (increase relative to reference group; p-values < 0.01) increased significantly more for urban-residing patients (44.9 relative to rural); Black, Asian, or Multi-Racial patients (Black: 52.1; Asian: 48.2; multi-racial: 57.5 relative to White), younger Veterans (age < 45: 113.0; age 45–64: 80.3 relative to age ≥ 65); and Veterans with major disabilities (127.3 relative to Veterans without special considerations).

**Conclusions:**

With the expansion of PTH telehealth during the pandemic, there was a shift in sociodemographic patterns among patients receiving at-home video-based care. Moving forward, VA may choose to test implementation strategies that target different demographic groups to support equitable access to PTH care.

**Supplementary Information:**

The online version contains supplementary material available at 10.1007/s44250-025-00256-0.

## Introduction

Telehealth has dramatically changed the landscape of healthcare in the last few years. While seemingly novel, telehealth, a term that encompasses both synchronous phone and video-based as well as asynchronous care has been around for many years. In various applications, telehealth has been shown to be safe, potentially equivalent to in-person care [1–3], and cost-effective [4]. It has been shown to increase access to care for groups at increased risk for health disparities [5].

The Veterans Health Administration (VHA) is the largest integrated health care system in the US and an early adopter of multiple telehealth modalities [6]. Like many health systems, the VHA has seen tremendous growth in the use of telehealth since the start of the COVID-19 pandemic [7–9]. However, while COVID drastically accelerated the pace of that growth, utilization of telehealth, particularly Provider-to-Home (PTH) telehealth was steadily increasing in VHA for several years before the pandemic began. Indeed, telehealth has been a critical element of the service offering for the VHA’s patient population for nearly two decades or longer. [10]

VHA is an equal access system that offers care throughout the US, without focus on provider reimbursement, making it a unique setting to examine health equity and disparities in care. While 93% of VHA patients live within a 40-mile drive of any VHA facility, only 26% live within a 40-mile drive of a VHA medical center with full specialty care, making telehealth an important component in VHA’s services [11]. As such, we sought to understand patterns of use of telehealth in VHA over the last several years in order to reflect on areas of strength and areas for improvement. We examined the uptake of telehealth by modality between October 2015 and March 2023. We further examined patterns of video-based care delivered in the home setting by patient race, rurality, age group, and VA priority status, during this time period.

Between 2015 and 2023, an average of 6.5 million patients were seen at the VHA annually across 171 medical centers and 1,112 outpatient sites in all 50 states (and U.S. territories). While the majority of VHA patients are White, VHA provides health care to large numbers of all racial minorities given its scale. For example, while American Indian or Alaska Native (AI/AN) Veterans constitute < 1% of the VHA population, they account for approximately 50,000 patients; similarly, while 85% of VHA patients are male, the remaining 15% include over 1 million female patients. The population of patients using the VHA is diverse with respect to age (20% < 45, 52% > 65), income (39% < $35,000/year) and rurality (33% rural/highly rural) [12, 13]. Finally, VHA patients are sicker on average than the general population in measures of both physical and mental health [14–16].

In VHA, the term “telehealth” refers to several modalities in use, including: (1) Synchronous Telehealth: care delivered in real time via video or telephone; and (2) Asynchronous Telehealth: also referred to as “Store and Forward” (SFT) Telehealth, this category consists of care delivered asynchronously using platforms that allow secure transmission of text, images, audio, or video between Veterans and clinicians or between multiple clinicians. Common examples of care delivered asynchronously in this manner include dermatology and radiology.

VHA has two broad subcategories of video visits: (1) Clinical Video Telehealth (CVT) visits are delivered between VHA healthcare facilities, and (2) Provider-to-Home (PTH) visits are delivered between a VHA provider and a Veteran directly to the Veteran’s home or other access point, including to a VHA provided mobile device or local area station for Veterans without access. VA Video Connect (VVC) is the proprietary platform on which VHA conducts these visits, but due to technological challenges, VHA has occasionally used other platforms (e.g., Doximity, Zoom) for such visits when necessary. This paper focuses on these aggregate PTH visits.

## Methods

### Population, measures and data sources

We used data from healthcare records of all Veterans seen at the VHA from October 2015 to March 2023, aggregated from VA’s Corporate Data Warehouse into its Pyramid analytics platform to compile quarterly telehealth Encounter and User counts by modality, and VHA’s yearly Unique Patient counts. For pre-post analyses, encounters only include PTH video visits completed between a VHA provider and the patient home or other (non-VA) access point. Users include VA patients who had at least one PTH encounter in the given quarter. Unique Patients include all patients who were seen at least once by VHA in any capacity throughout the Fiscal Year (FY). The government fiscal year begins October 1, so FY16 is from October 1, 2015, through September 30, 2016. Outcomes were defined as the number of Users with one or more telehealth encounters in a quarter per 1000 Unique Patients.

### Analysis

We graphically examined the use of telehealth by modality (CVT, PTH, and SFT) between October 2015 and March 2023. Although telephone is also a telehealth modality used in VHA, it was not a focus of this study and is thus excluded from our analysis.

Outcomes were broken down by race, rurality, age group, and VA priority group to examine rates of telehealth use in each group. For race, Veteran self-selections were used; Veterans who declined to answer (234,374 or approximately 3.4% of FY22 Unique Patients) or whose race was unknown (718,299 or approximately 10.5% of FY22 Unique Patients) were not included. For Rurality, VA categorizes Veteran locations as urban (Census tracts with at least 30 percent of the population residing in an urbanized area as defined by the Census Bureau), highly rural (sparsely populated areas—less than 10 percent of the working population commutes to any community larger than an urbanized cluster, which is typically a town of no more than 2,500 people), rural (land areas not defined as urban or highly rural) and insular islands based on their zip code [17]. We combined rural and highly rural populations and chose to exclude Veterans residing in Insular Islands as these represent a very small percentage (5,646 or < 0.1% of VHA patients). Age groups were defined as 44 and under, 45–64, and 65 and over. We collapsed VA priority groups (eligibility category for VHA benefits) into five strata: (1) Major disability- priority groups 1 and 4; (2) Minor disability- priority groups 2, 3, and 6; (3) Low income- priority group 5; (4) Low income with copays- priority group 7; (5) Veterans without special considerations- priority group 8 [19]. Veterans may qualify for more than one priority group, but are placed in the highest priority group (1 being highest, 8 being lowest priority) for which they are eligible. For a brief overview of VA priority groups, see Appendix 1.

We compared PTH utilization rates by sociodemographic groups (race, rurality, age group, and VA priority strata) before and after April 2020 (FY20Q3) using a pre-post analysis. Specifically, we used a fixed-effects linear regression to model the associations between each of the predictors (age, race, rurality, and priority group) and the outcome. Based on visual inspection of trends from October 2015 to March 2020, the parallel trend assumption appears to have been met for all stratifications. In addition to presenting visualizations of trends by strata, we report the regression estimates scaled to 1000 patients, 95% confidence intervals, and corresponding p-values. Analyses were performed using Stata, version 14 (StataCorp, LLC).

## Results

### Growth and proportion of encounters by modality

From October 2015 until the onset of COVID-19 in March 2020, quarterly telehealth encounters of all modalities and users per 1000 unique patients grew steadily, but PTH visits already significantly outgrew other encounters. In FY16Q1 (October-December 2015), there were approximately 28 telehealth users completing 11 SFT encounters, 28 CVT encounters, and 1.3 PTH encounters per 1000 unique patients in the VHA. By FY20Q2 (January-March 2020), there were 59 users completing 16 SFT, 38 CVT, and 34 PTH encounters; a change in proportion of PTH visits from 4.4% of all telehealth encounters to 46.7% during this period.

Beginning in FY20Q3 (the beginning of the COVID pandemic), there were significant changes in telehealth modalities used. In that quarter, CVT use decreased to 4.9 encounters per 1000 unique patients and has not yet recovered to pre-pandemic levels. SFT use also decreased initially but reached new highs by FY21Q2. Finally, PTH use expanded dramatically, averaging 348 quarterly encounters per 1000 unique patients in the post-pandemic period.

**Fig. 1:**
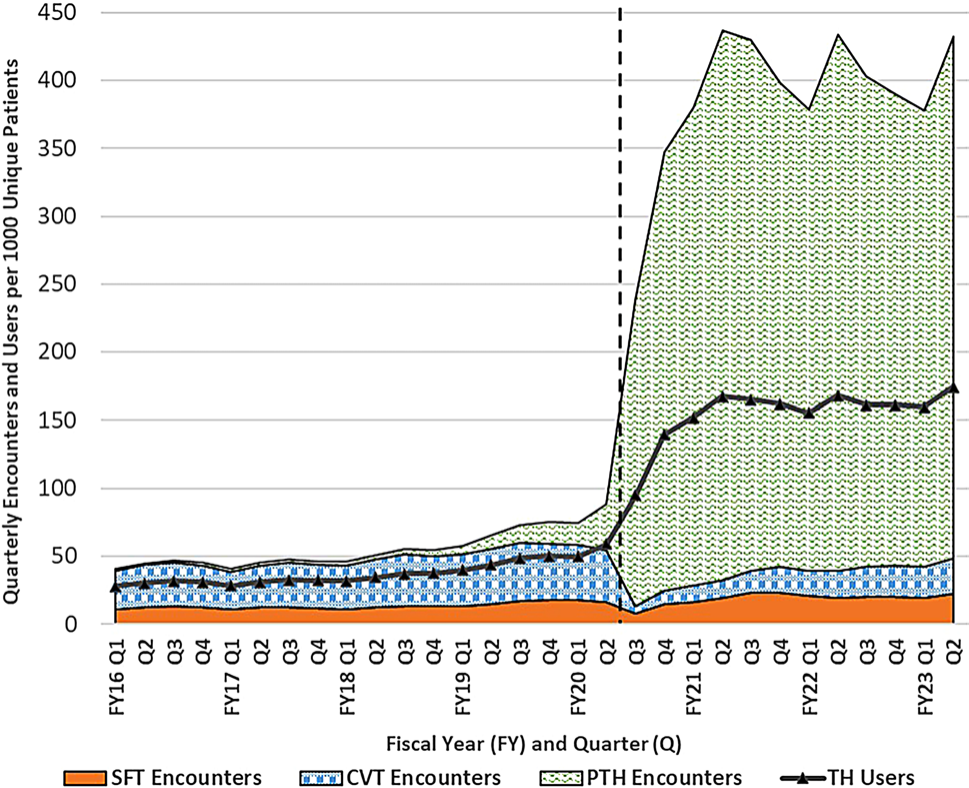
Quarterly Telehealth Encounters by Modality and Quarterly Users per 1000 Unique Patients in the Veterans Health Administration between 2015 and 2023. Outcomes measured included: Asynchronous Store and Forward Telehealth (SFT) encounters. Synchronous facility-to-facility Clinical Video Telehealth (CVT) encounters. Synchronous Provider To Home (PTH) Telehealth encounters. Users of any Telehealth (TH) modality. Dashed vertical line approximates March 11, 2020 (towards the end of FY20 Q2) when the World Health Organization declared COVID-19 a pandemic

Quarterly telehealth encounters also significantly outgrew quarterly telehealth users in the post-pandemic period, during which there were approximately 2.5 encounters (all modalities) per telehealth user, vs. 1.5 in the pre-pandemic period (Fig. 1) [18].

### Pre-post analyses

Table 1 summarizes results of our pre-post analyses by sociodemographic groups. Statistically significant increases in PTH use relative to referent groups are noted in bold and with asterisks.

**Table 1:**
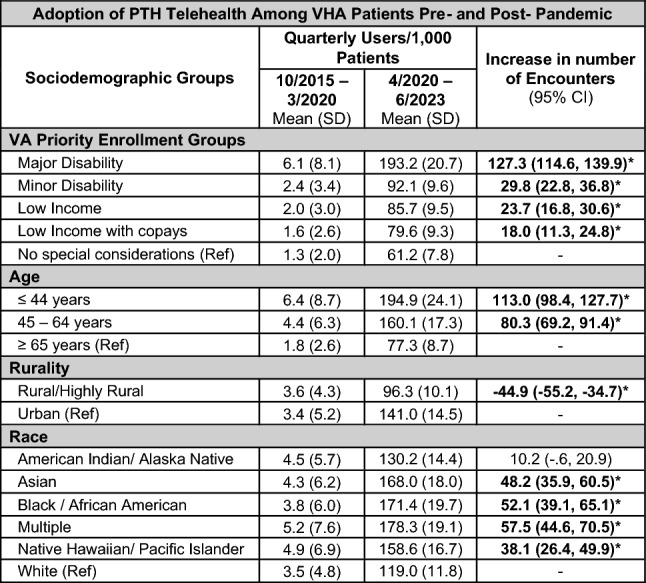
Results of our pre-post analyses by sociodemographic groups

*Statistically significant relative to referent group (Ref)

### PTH uptake by VA priority group

**Fig. 2:**
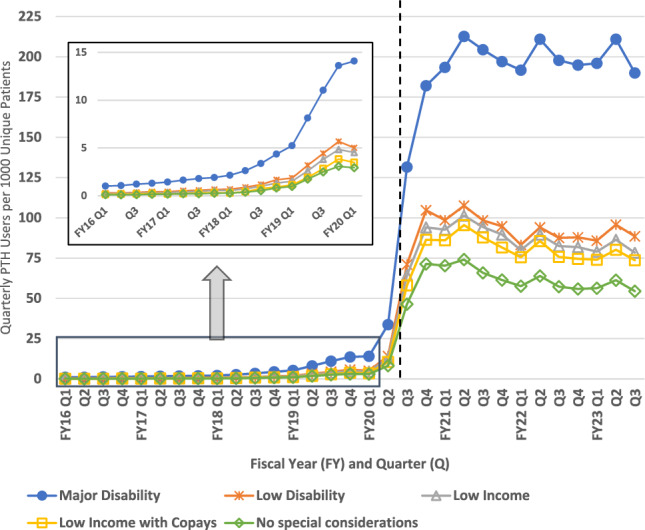
Quarterly Provider to Home (PTH) Users per 1000 Patients by Priority Stratus in the Veterans Health Administration between 2015 and 2023. Dashed vertical line approximates March 11, 2020 (towards the end of FY20 Q2) when the World Health Organization declared COVID-19 a pandemic

In the analysis of PTH uptake by priority group, the trends remained relatively consistent: Highly disabled Veterans were most likely to be users of PTH telehealth throughout both the pre- and post-pandemic periods. Of Veterans without service-related disabilities, those below the VA’s income threshold were initially more likely to be users, and the gap in uptake continued to grow. In the post-pandemic period, quarterly users per 1000 unique patients (increase relative to reference group; p-values < 0.01) increased significantly more for Veterans in all strata with special considerations (major disability: 127.3; minor disability: 29.8; low income: 23.7; low income with copays: 18.0). Lastly, uptake among highly disabled Veterans continues to grow, while other groups appear to have plateaued (Fig. 2).

### PTH uptake by age group

**Fig. 3:**
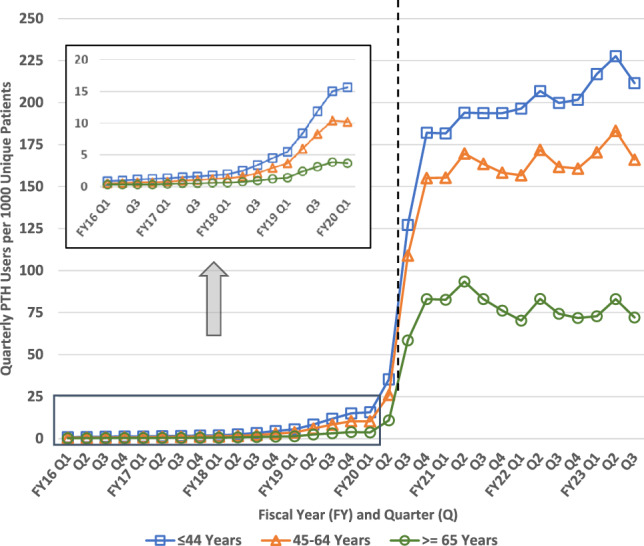
Quarterly Provider to Home (PTH) Users per 1000 Patients by Age Group in the Veterans Health Administration between 2015 and 2023. Dashed vertical line approximates March 11, 2020 (towards the end of FY20 Q2) when the World Health Organization declared COVID-19 a pandemic

In the analysis of PTH uptake by age group, the trends were again consistent. Veterans aged 44 and younger were most likely to have PTH visits in each quarter pre- and post-pandemic, followed by those aged 45–64, followed by those 65 and older. In the post-pandemic period, quarterly users per 1000 unique patients increased significantly more for patients in the two younger groups (age < 45: 113.0; age 45–64: 80.3 relative to age ≥ 65) (Fig. 3).

### PTH uptake by rurality

**Fig. 4:**
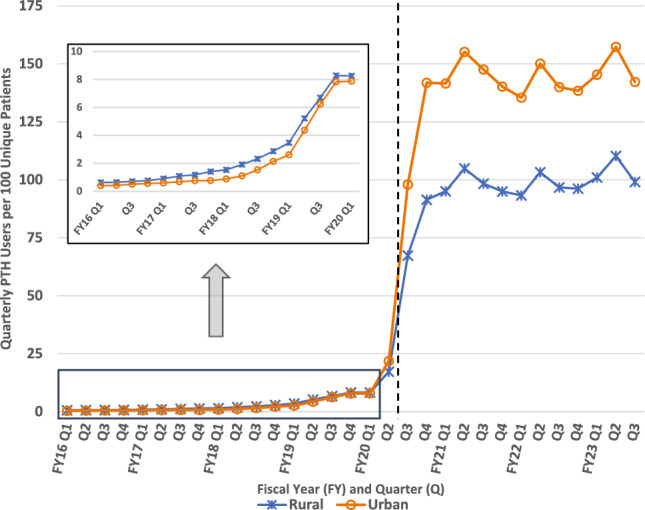
Quarterly Provider to Home (PTH) Users per 1000 Patients by Rurality. Dashed vertical line approximates March 11, 2020 (towards the end of FY20 Q2) when the World Health Organization declared COVID-19 a pandemic

Until FY20Q2, rural Veterans were more likely than urban Veterans to utilize PTH visits in every single quarter. In the post-pandemic period, quarterly users of PTH encounters increased significantly more for urban patients (44.9 relative to rural); urban patients had higher rates of usership in every single quarter beginning FY20Q2 and reached uptake of 157 Users per 1000 Unique Patients, or 15.7%, compared to a high of only 11.0% of rural patients (Fig. 4). In this analysis, between group trends reversed notably at the beginning of the pandemic but remained consistent both before and after.

### PTH uptake by race

When examining uptake of PTH visits by race, we observed shifts among demographic groups earlier than in other demographic analyses. Until Q4 of FY18, AI/AN Veterans were nearly always the most likely to utilize PTH video visits compared to patients of other races. Starting the following quarter (one and a half years before the COVID pandemic began) the racial makeup of PTH encounters fluctuated as overall usership began to increase at a faster pace.

**Fig. 5:**
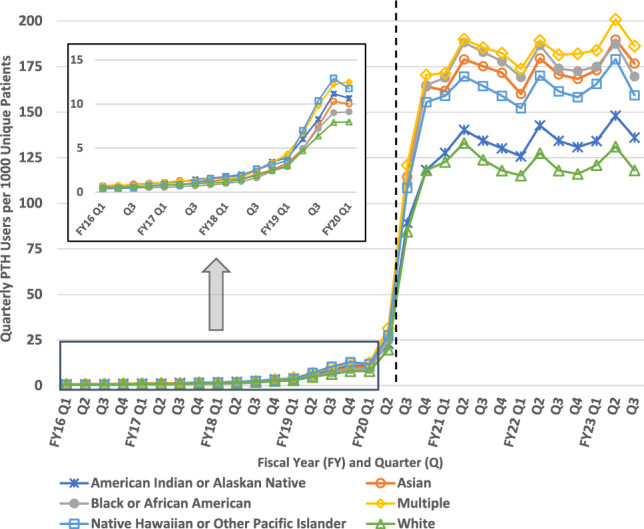
Quarterly Provider to Home (PTH) Users per 1000 Patients by Race. Dashed vertical line approximates March 11, 2020 (towards the end of FY20 Q2) when the World Health Organization declared COVID-19 a pandemic

In the post-pandemic period, we observed significant differences in PTH uptake among racial demographic groups that have lasted as PTH expansion plateaued in 2022. While all subgroups experienced increased usership, quarterly users per 1000 patients increased significantly more for multi-racial, Black, Asian, and NH/PI Veterans (multi-racial: 57.5; Black: 52.1; Asian: 48.2; Native Hawaiian and Pacific Islander: 38.1 relative to White) than White and AI/AN patients, respectively (Fig. 5). Further, from October 2021 through June 2023 (the most recent quarter measured), there have been no changes in between group trends for any racial group.

## Discussion

While there was a modest but steady increase in all types of telehealth use within VHA between October 2015 and March 2020, the COVID-19 pandemic spurred rapid and asymmetrical expansion and adoption of these services. The pandemic prompted a shift in modalities of telehealth delivery, as PTH video visits became more widely available, desirable, and most importantly, necessary (due to restrictions on in-person in-clinic visits) to Veterans. As PTH visits became a necessity, there was a concurrent shift in the demographics of Veterans using them. Before the pandemic, Veterans most likely to be PTH users per capita were rural residing, and AI/AN or multi-racial; since the pandemic they have been urban residing and Asian, multi-racial, or Black. While younger Veterans and those in highly disabled priority groups were the most likely to be PTH users throughout the study period, these trends widened during the pandemic.

The changes observed in demographics of PTH users pre- and post-pandemic make sense. In the pre-pandemic period, rural and/or disabled Veterans were more likely beneficiaries of PTH visits due to the distance between them and their nearest VHA facilities, difficulty driving, mobility issues, and other challenges. While PTH telehealth was beginning to become popularized during this period, these encounters were mainly used in populations with the highest physical barriers to in-person access. The small percentages of even these patient populations represented those with access to facilitators such as a broadband internet connection, a higher level of digital literacy, or myriad others cited in both VA and non-VA research literature [20–23].

The COVID-19 pandemic catalyzed a new form of telehealth in VHA. This includes services such as entirely remote mental health and telehealth-first primary care that is complemented by office or home visits [23, 24]. Beginning in April 2020, we observed a rapid increase in PTH encounters in all groups, significantly among younger, racially diverse, urban populations that were not the traditional target audience of telehealth interventions. Importantly, the shift in telehealth encounters towards PTH, increase in PTH use among all demographic groups, and gaps in uptake between demographic groups have all persisted long after the most acute societal impacts of the pandemic subsided. This may indicate that PTH care has gone from a stopgap addressing the cancellation of many non-emergent in-clinic appointments, to a preferred modality of care in certain scenarios, but that barriers to PTH care have not yet been addressed.

Our data do not elucidate the barriers impeding PTH uptake among the populations left behind during this transition. However, during the necessitation of telehealth encounters in 2020–21, certain populations may have had their care converted into PTH much more readily than others. Among VHA patients, research has identified populations with greater video telehealth use consistent with our findings, including Veterans residing in urban areas, younger Veterans, Veterans with disabilities, and Veterans with lower family incomes [9, 25].

Our findings are also consistent with several studies in the general US population and among Veterans, which have identified similar demographic groups, including rural and older populations, at greater risk of digital disparities such as limited access to video enabled devices or broadband internet [23, 33–35]. Studies on the general population have also shown digital literacy to be a major barrier to video telehealth use in older adults [22]. Findings on digital inclusion diverge between VA and non-VA populations, however, along racial and socio-economic lines. Research on the general population has identified subgroups at greater risk of digital disparities that were inconsistent with our findings in these demographic analyses [26–29, 33, 35]. Our data showed that Black Veterans and non-disabled Veterans with family income below the means threshold were more likely than their counterparts (White Veterans and non-disabled Veterans with a family income above the means threshold, respectively) to be users of PTH video visits. This contrasts with studies in the general population, which have found that Black and lower income families were less likely to own video-enabled devices or have broadband internet connection, and Black patients specifically to be more reliant on telephone than video for telehealth visits. Reasons for these inconsistencies may include VA policies on telehealth coverage, patient financial responsibilities, and interventions focused on equal access, such as the VA’s loaned tablet program [7]. Our findings may also be skewed by the overlap of subgroups within the VA population. Our aggregate data is likely confounded by demographic characteristics demonstrated to be strong confounders of telehealth use, including age, sex, and rurality. Black Veterans may be younger, more likely to reside in urban areas, and/or have more service-connected disabilities on average than their white counterparts. In a study of Veteran’s utilization of video care in the first year of the pandemic, after adjusting for patient demographics like age, sex, and rurality, Black Veterans were only slightly more likely to have a video visit than White Veterans (OR 1.02 (95% CI 1.01, 1.02) [9]. Another study found low-income Veterans less likely to engage in telehealth, but that once they did engage, they had higher rates of telehealth use [30]. Lastly, Veterans with higher incomes are more likely to have other primary sources of healthcare coverage (e.g., employer sponsored) [9, 31, 32]. This greater reliance on VA care compared to Veterans enrolled without special considerations may explain the higher rate of telehealth use among low income Veterans seen in our data.

### Limitations

Our study was limited by several factors. Analyses are unadjusted and did not examine interaction effects between more than one factor. Additionally, there is a sizable amount of demographic data missing from our analysis; each year, approximately 175,000–200,000 Unique Patients declined to self-identify their race, and another 500,000–750,000 were marked “Unknown” in the summary data we accessed. Veterans who declined to self-identify race and Veterans who had unknown values for race tend to be lower utilizers of VHA care, including telehealth [9], as race is collected during VHA encounters. Finally, our study utilized repeated cross-sectional data, and thus our analyses for this paper do not distinguish between PTH visits of different patients between quarters versus the same patients receiving multiple PTH visits.

## Conclusion

This study provides valuable data on demographics of PTH visits in the VA over a 7 year period. Our results affirm previous findings that rural and older Veterans continue to experience a “digital divide” in access to and engagement with telehealth resources. While much evidence exists about the populations at risk and barriers they face, far fewer studies have tested interventions to increase PTH video telehealth usership in these populations. As an equal access system without focus on provider reimbursement, VA is not only an outstanding setting to examine health equity and disparities in PTH utilization, but also one in which to test interventions and implementation strategies aimed at addressing identified disparities. Moving forward, VA may choose to test implementation strategies that target different demographic groups to support equitable access to PTH video care.

## Supplementary Information

Below is the link to the electronic supplementary material.

Supplementary file1 (DOCX 18 KB)

## Data Availability

Data sharing statement Due to US Department of Veterans Affairs (VA) regulations and our ethics agreements, the analytic data used for this evaluation are not permitted to leave the VA firewall without a Data Use Agreement. VA data are made freely available to VA authorized researchers with an approved VA protocol. For more information, please visit https://www.virec.research.va.gov or contact the VA Information Resource Center at VIReC@va.gov.
