# Supplementary material for: Sociodemographic patterns of provider-to-home telehealth use within the Veterans Health Administration between 2015 and 2023
Source: Discov Health Syst. 2025 Jul 9;4(1):80. doi: 10.1007/s44250-025-00256-0 (PMC12241211; doi:10.1007/s44250-025-00256-0)
Supplement: Supplementary file 1 — Supplementary file1 (DOCX 18 KB) [file 44250_2025_256_MOESM1_ESM.docx]

**Appendix 1: Additional Definitions and Methods**

**VA Fiscal Years (FY):** VA FYs begin on October 1 of the previous year, i.e., FY24 began on October 1, 2023 and will conclude on September 30, 2024.

**VA Priority Strata:** VA priority groups are eligibility categories for VHA benefits based on military service history, disability rating, income level, qualifying for Medicaid, and other benefits received. A disability could be any medical problem judged to be attributable to a Veteran’s service in the military and thus could include traumatic brain injury but could also include diabetes or heart disease. There may be overlap in eligibility for priority groups – i.e., a Veteran with a service-related disability may also qualify for a low income priority group. Veterans are placed in a group based on their highest priority rating (1 being highest and 8 being lowest priority). We collapsed the eight VA priority groups into five strata:

- Major Disability – Veterans in priority groups 1 (>50% service-connected disability) and 4 (VA catastrophically disabled)
- Low Disability – Veterans in priority groups 2 (30-40% service-connected disability), 3 (10-20% service-connected disability, discharged due to disability), and 6 (military exposures and recent combat Veteran)
- Low Income – Veterans in priority group 5 (annual income below area-adjusted Means Threshold)
- Low Income with Copays – Veterans in priority group 7 (annual income below Means Threshold, who voluntarily pay copays)
- No Special Considerations – Veterans in priority group 8

**Rurality:** VA categorizes Veteran locations as urban (Census tracts with at least 30 percent of the population residing in an urbanized area as defined by the Census Bureau), highly rural (sparsely populated areas – less than 10 percent of the working population commutes to any community larger than an urbanized cluster, which is typically a town of no more than 2,500 people), rural (land areas not defined as urban or highly rural) and insular islands based on their zip code. We combined rural and highly rural populations and chose to exclude Veterans residing in Insular Islands as these represent a very small percentage (5,646 or <.1% of VHA patients).
